# Genome-wide association study for seedling heat tolerance under two temperature conditions in bread wheat (*Triticum aestivum* L.)

**DOI:** 10.1186/s12870-024-05116-2

**Published:** 2024-05-21

**Authors:** Chao Fu, Ying Zhou, Ankui Liu, Rui Chen, Li Yin, Cong Li, Hailiang Mao

**Affiliations:** National Key Laboratory of Crop Genetic Improvement, Huazhong Agricultural University, Wuhan, 430070 China

**Keywords:** Heat stress tolerance, GWAS, QTL, Seedling stage, *Triticum Aestivem* L.

## Abstract

**Background:**

As the greenhouse effect intensifies, global temperatures are steadily increasing, posing a challenge to bread wheat (*Triticum aestivum* L.) production. It is imperative to comprehend the mechanism of high temperature tolerance in wheat and implement breeding programs to identify and develop heat-tolerant wheat germplasm and cultivars.

**Results:**

To identify quantitative trait loci (QTL) related to heat stress tolerance (HST) at seedling stage in wheat, a panel of 253 wheat accessions which were re-sequenced used to conduct genome-wide association studies (GWAS) using the factored spectrally transformed linear mixed models (FaST-LMM). For most accessions, the growth of seedlings was found to be inhibited under heat stress. Analysis of the phenotypic data revealed that under heat stress conditions, the main root length, total root length, and shoot length of seedlings decreased by 47.46%, 49.29%, and 15.19%, respectively, compared to those in normal conditions. However, 17 varieties were identified as heat stress tolerant germplasm. Through GWAS analysis, a total of 115 QTLs were detected under both heat stress and normal conditions. Furthermore, 15 stable QTL-clusters associated with heat response were identified. By combining gene expression, haplotype analysis, and gene annotation information within the physical intervals of the 15 QTL-clusters, two novel candidate genes, *TraesCS4B03G0152700/TaWRKY74-B* and *TraesCS4B03G0501400/TaSnRK3.15-B*, were responsive to temperature and identified as potential regulators of HST in wheat at the seedling stage.

**Conclusions:**

This study conducted a detailed genetic analysis and successfully identified two genes potentially associated with HST in wheat at the seedling stage, laying a foundation to further dissect the regulatory mechanism underlying HST in wheat under high temperature conditions. Our finding could serve as genomic landmarks for wheat breeding aimed at improving adaptation to heat stress in the face of climate change.

**Supplementary Information:**

The online version contains supplementary material available at 10.1186/s12870-024-05116-2.

## Background

Bread wheat (*Triticum aestivum* L.) is the most widely cultivated cereal crop and serves as a staple food for over 35% of the global population [1, 2]. The ideal temperature range for wheat growth is around 22/14℃ (day/night) [3]. With the increasing impact of climate change, particularly under hot environments, wheat production is at high risk [4]. It is shown that for every 1℃ increases in ambient temperature, there is a significant decrease in wheat yield, ranging from 4.1 to 6.4% [5, 6]. Hence, there is an urgent need to unravel the genetic networks linked to heat stress perception and adaptation in crops. This will aid in developing crop varieties with enhanced thermotolerance through molecular breeding techniques.

Heat stress causes diverse negative effects during the growing process of wheat [7]. When exposed to high temperature during the anthesis stage, the number of spikelets per panicle will dramatically decline and the grain filling rate will also be seriously affected [8, 9]. High temperature was also found to cause a lower photosynthetic rate and accelerating the senescence of leaf, which resulted in largely reduced grain yield per plant [10–13]. Moreover, the levels of both protein and starch are reduced when the temperature is over 30℃ [14–16].

Various parameters have been employed to assess HST in wheat, including yield, number of panicles, number of grains per panicle, thousand grain weight (TGW), soluble sugar content, canopy temperature, chlorophyll content, chlorophyll fluorescence parameters, activity of antioxidant enzymes, and degree of membrane damage [17–25].

Genome-wide association studies (GWAS) and quantitative trait loci (QTL) mapping have identified genetic loci associated with HST across all 21 chromosomes of wheat [26–28]. For instance, ten QTLs associated with leaf chlorophyll content, canopy temperature, thousand-grain weight, grain yield heading and maturity were found in a set of 166 doubled haploid lines under late sown conditions, and these QTLs could be targeted for genetic improvement and marker-assisted selection for heat tolerance in wheat [29]. Twenty-seven single nucleotide polymorphism markers (SNPs) and two putative *MIP1-like* genes (*TraesCS6A02G124100* and *TraesCS6D02G114400*) related to HST traits were identified in another study [30]. In addition, a stable QTL of photosynthetic rate was detected on Chr2D in a recombinant inbred line population [19]. In a GWAS study with 688 winter wheat using the wheat 90 K array, ten QTLs were identified on chromosomes 1B, 2B, 3 A, 3B, 5 A, 5B, and 7D associated with HST [31]. Thirty-three high-confidence stable QTL and three candidate genes, including *TaELF3*, which enhanced the adaptability of accessions to heat stress, were discovered based on grain-related traits of spring wheat and the iSelect 90 K array [32]. Furthermore, two wheat HST loci, *TaHST1* and *TaHST2*, were fine-mapped to narrow intervals on chromosomes 4AL and 4DS, respectively, and were found to play crucial roles in maintaining wheat vegetative and reproductive growth under high temperature [33, 34]. These loci have shown promising potential target for improving HST in wheat.

Cloning genes associated with HST is a challenging task that requires substantial time and effort to ascertain heat response phenotypes [35]. Although researchers have streamlined methods using seedling heat response phenotypes such as root length (RL), shoot length (SL), and root weight (RW), the correlation between HST at the seedling stage and the adult stage has not been confirmed [36]. However, recent research has shown promising results regarding the variation in root lengths among wheat genotypes under heat stress [37]. They found that HST varieties exhibited longer seedling roots compared to susceptible ones, and these HST varieties also demonstrated higher resistance at the adult stage after heat treatment, as indicated by measurements of chlorophyll content and yield. Additionally, there was a significant positive relationship between heat tolerance at the seedling stage and the adult stage (*r* = 0.6930), suggesting the possibility of early selection at the seedling stage for breeding heat tolerance [37].

The objectives of this study were as follows: (i) to measure heat response phenotypes and assess heat tolerance in a diverse range of wheat varieties at the seedling stage, (ii) to identify genetic loci associated with HST through GWAS, and (iii) to predict candidate genes involved in heat stress tolerance.

## Results

### Phenotypic variation

**Fig. 1:**
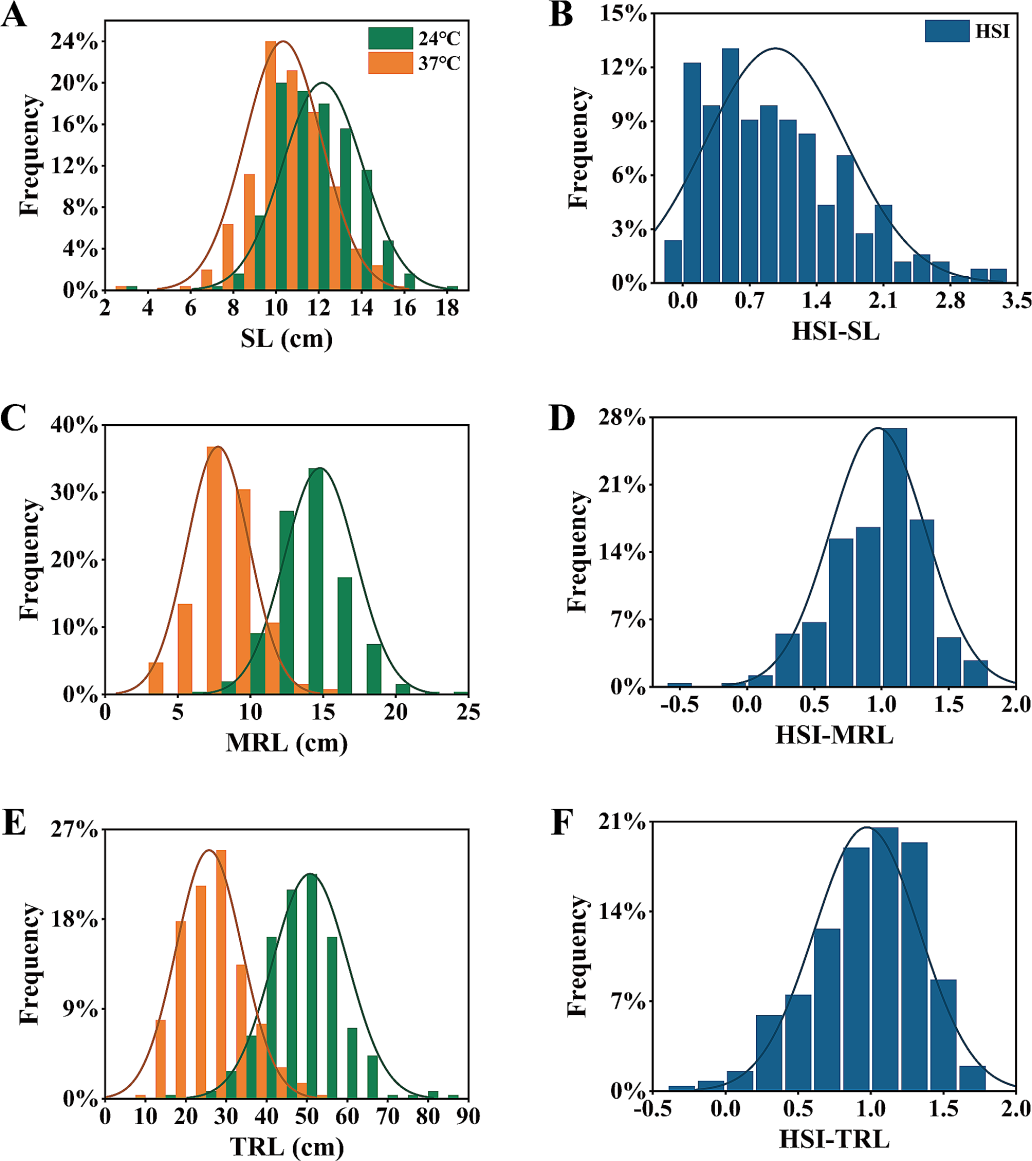
Frequency distribution of different traits (A, C and E) showed shoot length (SL), main root length (MRL), and total root length (TRL) at normal (green) and heat stress (orange) conditions, while (B, D and F) showed HSI of shoot length (HSI-SL), main root length (HSI-MRL), and total root length (HSI-TRL), respectively

**Fig. 2:**
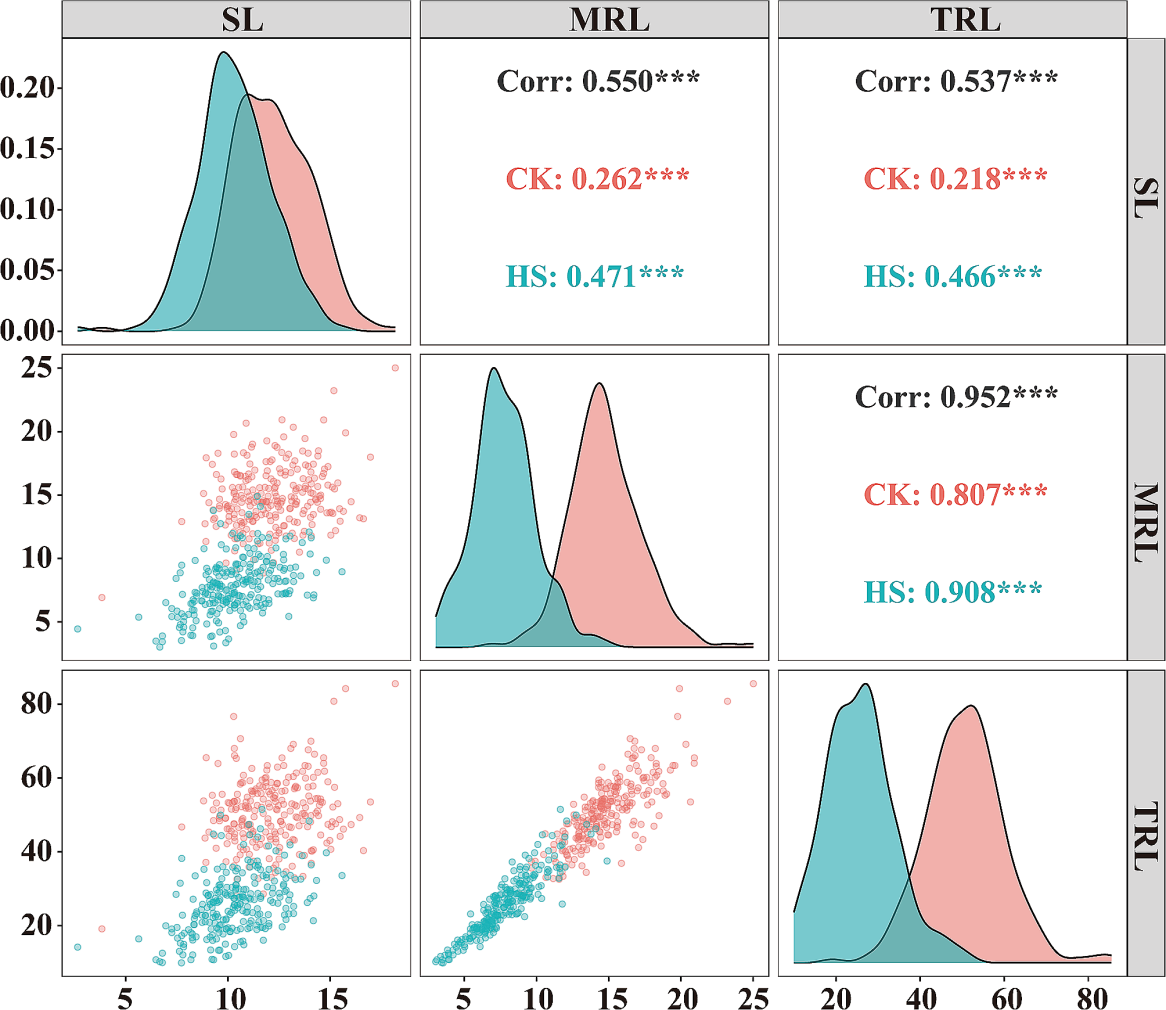
Pearson’s correlation coefficients describing association of various traits in wheat under normal and heat stress conditions

Continuous variation was observed for all tested traits among the 253 wheat accessions grown under both normal and high temperature conditions (Table 1; Fig. 1). Under normal condition, the average MRL was 14.79 cm, ranging from 6.93 to 25.02 cm, which was longer compared to the average grown under 37℃ (with average of MRL 7.77 cm, range: 3.02 to 14.89 cm). The average TRL, which was calculated the total length of all the roots from each plant, was significantly decreased by 47.45% under high temperature compared to normal condition. The average SL at high temperature was 10.33 cm, ranging from 2.65 to 15.58 cm, while at normal temperature, the average was 12.17 cm, with a range of 3.84 to 18.19 cm. Overall, all investigated traits were highly impacted by high temperature, with a negative effect observed in most tested accessions. Pearson correlation coefficients were calculated to assess the relationships between MRL, TRL, and SL under normal and high temperature conditions (Fig. 2). Under normal treatment, there was a correlation (*r* = 0.262, *P* < 0.001) between SL and MRL. Similarly, a positive correlation (*r* = 0.471, *P* < 0.001) was observed between SL and MRL under high temperature. The correlations between SL and TRL under normal (*r* = 0.218, *P* < 0.001) and high temperature (*r* = 0.466, *P* < 0.001) were consistent with the relationships between SL and MRL. As expected, a significant and strong correlation was found between MRL and TRL under both normal (*r* = 0.807, *P* < 0.001) and high temperature (*r* = 0.908, *P* < 0.001) conditions.

**Table 1:** Phenotype statistics analysis of seedling traits under normal and heat stress condition

Traits ^a^	Control				HS ^c^				HSI ^d^		
	Mean (cm)	Range (cm)	SD ^b^		Mean (cm)	Range (cm)	SD		Mean (cm)	Range (cm)	SD
MRL	14.79	6.93–25.02	2.46		7.77	3.02–14.89	2.14		0.98	0.06–1.69	0.34
TRL	50.78	19.15–85.54	9.36		25.75	9.95–51.50	8.14		0.99	0.08–1.68	0.34
SL	12.17	3.84–18.19	1.87		10.33	2.65–15.58	1.80		0.97	-0.03-3.33	0.73

^a^ MRL, main root (the longest root) length; TRL, total root length; SL, shoot length

^b^ SD, standard deviation

^c^ HS, heat stress

^d^ HSI, heat susceptible index

The heat susceptible index (HSI) is widely used to evaluate HST for wheat [1, 37]. The wheat varieties were considered to be high heat-resistance materials if HSI were less than 0.5 [1]. Due to the strong relationship between HSI of seedling root length and heat tolerance at the adult stage [37], HSI of MRL (HSI-MRL) and TRL (HSI-TRL) were used as standards to identify HST varieties. Accordingly, 17 varieties were identified to be resistant to high temperature. White Saidi, Een 1, and Jinan 8 showed extremely low HSI values and are considered heat-tolerant strains that can be used for heat-tolerance breeding in wheat (Table S3).

### SNP marker coverage and genetic diversity

**Fig. 3:**
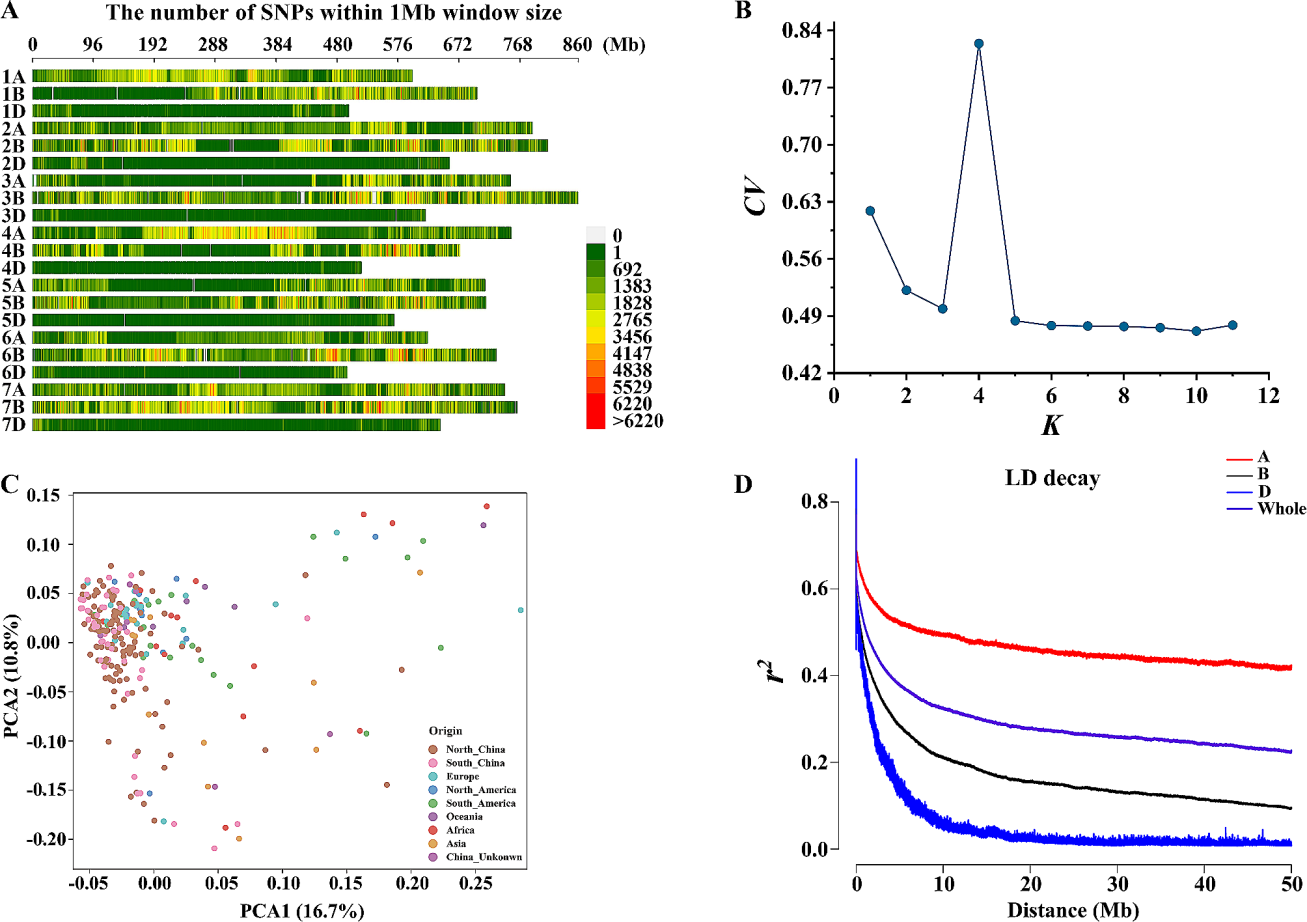
Genetic diversity and population structure of the wheat association panel (A) The distribution of SNPs with minor allele frequency larger than 0.95 and missing rate ≥ 80% (B) Plot of cross-validation (*CV*) error against putative *K* ranging from 1 to 11 (C) Principal component analysis of 253 accessions. Proportions of explained variance of principal components (PCs) 1 and 2 are indicated on the axes. Different colours represent geographical origin of wheat lines (D) Linkage disequilibrium (LD) decay for the whole genome and A, B and D subgenomes. Decay curves of A, B, D and whole genome were presented in squared correlation of allele frequencies at diallelic loci (*r*^*2*^)

From 253 re-sequenced wheat accessions, 13,116,299 high-quality SNP markers were filtered for subsequent analysis [38, 39]. Out of that, 5,415,520 SNPs were detected on the A sub-genome, 7,121,654 SNPs on the B sub-genome, and 579,125 SNPs on the D sub-genome. The physical map lengths for these sub-genomes were 4,975.29 Mb, 5,256.89 Mb, and 4,001.36 Mb, respectively (Table S4). Chr4D had the fewest SNPs (37,818), while Chr7B had the most SNPs (1,324,537). The SNP diversity values for each chromosome ranged from 72.96 per Mb to 1,733.51 per Mb, with an average of 921.51 per Mb (Fig. 3A; Table S4).

### Population structure and linkage disequilibrium

In the ADMIXTURE analysis, 253,963 SNP markers and 253 wheat accessions were used to analyze the population structure. The cross-validation error (*CV*) was high with large variation for *K* values less than 5, but the *CV* error became steady for *K* values between 5 and 11 (Fig. 3B). Therefore, *K* = 5 was considered as the optimal value, and the panel was split into 5 sub-populations (Fig. S2).

Principal component analysis (PCA) showed moderate variation, with PC1 explaining 16.7% of the variation and PC2 explaining 10.8% of the variation. The landraces were separated from cultivars, and the varieties from different wheat regions were also distinct from each other (Fig. 3C; Fig. S3). Similarly, the neighbor-joining tree and kinship analyses indicated that there was no conspicuous population classification, with rare exceptions in Chinese wheat strains (Fig. S4, A-B). Overall, the results indicated a high genetic diversity of the tested panel.

Linkage disequilibrium (LD) analysis was performed using all the SNPs. The LD decay in the D sub-genome dropped more quickly, with the r^2^ value dropping to 0.45 (half of the initial value) at 0.59 Mb. In the B sub-genome and the whole genome, the LD dropped to 0.50 at 2.37 Mb and 4.29 Mb, respectively. Among the whole genome, the A sub-genome experienced slowest rates of the LD decay than that in the B and D sub-genomes, with the LD dropping to 0.50 at 8.22 Mb (Fig. 3D). Combined with previous research [40, 41], a physical distance of 3 Mb was chosen as the mean genetic unit in the following analysis.

### Genome-wide association analysis and QTL identification

**Fig. 4:**
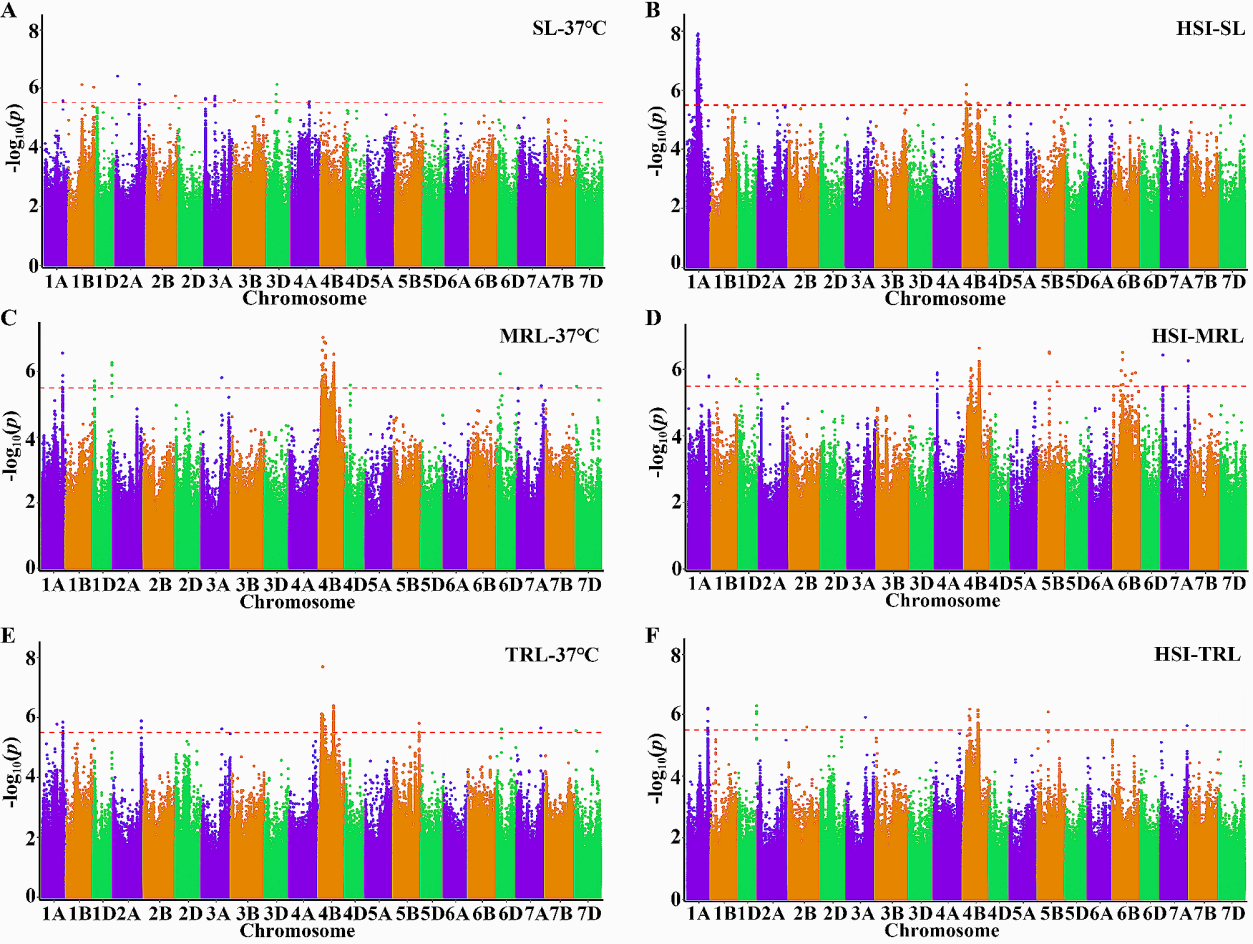
Manhattan plots showing density of SNP markers associated with different traits The x axis represents chromosomes and y axis refers to -log10(p) for different traits. Red dash line indicates the threshold of genome-wide significant *P*-value (1 × 10 − 5.5). Manhattan plots for shoot length (SL) (A), main root length (MRL) (C), total root length (TRL) (E) under 37℃ conditions and HSI for SL (B), MRL (D) and TRL (F)

A total of 1012 MTAs (marker-trait associations) were identified under two conditions (Fig. 4; Fig. S5; Table S5). The numbers of significant SNPs for each trait ranged from 17 for MRL-CK to 446 for HSI-SL. These MTAs formed 115 QTLs based on the previously defined mean genetic unit and were distributed on all chromosomes except for Chr2D (Table 2; Table S6). Among them, 38 QTLs were identified under normal condition, including 12 for MRL-CK, 16 for TRL-CK, and 10 for SL-CK, while 77 QTLs were detected under heat condition, including 13 for MRL-HS, 16 for TRL-HS, 13 for SL-HS, and 39 for HSI (Table 2; Table S6). Some QTLs (such as *Qhmrl.hzau-4B.1*, *Qhtrl.hzau-4B.1*, *Qhmrl.hzau-4B.3*, *Qhtrl.hzau-4B.4*, *Qhmrl.hzau-4B.5*, and *Qhsi.hzau-4B.11*) had a larger number of significant SNPs and were also linked to HST, suggesting their potential involvement in the response to high temperature stress (Table S6). *Qhsi.hzau-1 A.2* had the most significant SNPs (427), but it was only detected for HSI-SL.

**Table 2:** Number of MTAs and QTLs identified for nine traits by GWAS

Traits ^a^	MTAs ^b^	QTLs ^c^	Sub-genome ^d^		
			A	B	D
SL-CK	24	10	4	5	1
MRL-CK	18	12	4	6	2
TRL-CK	34	16	5	9	2
SL-HS	20	13	6	4	3
MRL-HS	168	13	3	5	5
TRL-HS	140	12	5	5	2
HSI-SL	446	10	6	4	0
HSI-MRL	123	20	4	14	2
HSI-TRL	39	9	3	5	1
Total	1012	115	40	57	18

^a^ SL-CK, MRL-CK, and TRL-CK, the shoot length, main root length, and total root length under normal condition; SL-HS, MRL-HS, and TRL-HS, the shoot length, main root length, and total root length under heat stress environment; HSI-SL, HSI-MRL, and HSI-TRL, heat susceptible index of shoot length, of main root length and of total root length

^b^ The number of significant marker-trait associations (MTAs) identified by GWAS.

^c^ The number of identified QTLs through GWAS

^d^ The number of QTLs in each sub-genome

Fifty-two QTLs detected for more than one traits were considered as stable QTLs (Table 3). These QTLs were further categorized into 17 QTL-clusters, including 15 clusters related to HST (designated *QHST1*-*QHST15*) detected under heat conditions while 2 clusters detected under normal conditions related to root length (designated *QRL1* and *QRL2*). Interestingly, some QTL-clusters could be detected both in shoot and root, such as *QHST1*, *QHST6*, *QHST7*, and *QHST9* among the 15 QTL-clusters detected under heat conditions, hinting the universal response to high temperature stress in different organs of wheat. In addition, some of the HST-related QTL-clusters overlapped with QTLs related to high temperature stress at flowering stage, grain filling stage, and maturation stage [30, 42–48] (Table 3), suggesting their validity across different growth stages in wheat.

**Table 3:** The QTL-clusters divided based on the physical distance of the QTLs

QTL clusters ^a^	QTLs	Traits	Chr	Position (Mb)	QTLs/loci reported ^b^	
*QHST1*	*Qhtrl.hzau-1 A.1*	TRL-HS	1 A	368.78-376.34	*QYld.agt-SG.1 A*	[42]
	*Qhsi.hzau-1 A.5*	HSI-SL				
*QHST2*	*Qmrl.hzau-1 A*	MRL-CK	1 A	519.30-530.39	QGNP-HS-R1	[30]
	*Qhmrl.hzau-1 A*	MRL-HS				
	*Qhtrl.hzau-1 A.2*	TRL-HS			*QHknm.tam-1 A*	[43]
	*Qhsi.hzau-1 A.6*	HSI-TRL				
	*Qhsi.hzau-1 A.7*	HSI-MRL				
*QHST3*	*Qhmrl.hzau-1D.2*	MRL-HS	1D	470.35-476.36	*AX-179,558,207* (SW)	[1]
	*Qhsi.hzau-1D.2*	HSI-TRL			*AX-94,752,800* (TKW-DHS)	[30]
	*Qhsi.hzau-1D.3*	HSI-MRL				
*QHST4*	*Qsl.hzau-2 A.1*	SL-CK	2 A	55.49–65.21	*QSRHS.nri-2 A*	[36]
	*Qhsl.hzau-2 A.1*	SL-HS				
*QHST5*	*Qhmrl.hzau-3 A*	MRL-HS	3 A	516.86-522.86	*QTgw.agt-SG.3 A*	[20]
	*Qhtrl.hzau-3 A*	TRL-HS				
*QHST6*	*Qhmrl.hzau-4B.1*	MRL-HS	4B	63.92- 115.19	*QCMS-4B*	[44]
	*Qhtrl.hzau-4B.1*	TRL-HS			*AX-111,251,816* (GNP-HS)	[30]
	*Qhsi.hzau-4B.1*	HSI-SL				
	*Qhsi.hzau-4B.2*	HSI-SL			*Tdurum_contig28490_463* (DM)	[45]
	*Qtrl.hzau-4B.1*	TRL-CK				
*QHST7*	*Qhmrl.hzau-4B.2*	MRL-HS	4B	141.89-190.03	*GENE-2129_76-RAC875_c45747_87* (DM)	[45]
	*Qhtrl.hzau-4B.2*	TRL-HS				
	*Qhsi.hzau-4B.3*	HSI-MRL				
	*Qhsi.hzau-4B.3*	HSI-TRL				
	*Qhsi.hzau-4B.5*	HSI-SL				
	*Qhsi.hzau-4B.6*	HSI-MRL				
	*Qhmrl.hzau-4B.3*	MRL-HS				
	*Qhsi.hzau-4B.7*	HSI-TRL				
	*Qhtrl.hzau-4B.3*	TRL-HS				
*QHST8*	*Qhmrl.hzau-4B.4*	MRL-HS	4B	345.52-359.15		
	*Qtrl.hzau-4B.2*	TRL-CK				
	*Qhsi.hzau-4B.9*	HSI-MRL				
*QHST9*	*Qhmrl.hzau-4B.5*	MRL-HS	4B	369.58-396.59	*AX-94,606,541* (NDVI)	[46]
	*Qhtrl.hzau-4B.4*	TRL-HS			*S4B_370054947* (NDVI)	[47]
	*Qhsi.hzau-4B.10*	HSI-TRL				
	*Qhsi.hzau-4B.11*	HSI-MRL			*S4B_396518901* (MT)	[47]
	*Qhsi.hzau-4B.12*	HSI-SL				
*QHST10*	*Qhsi.hzau-5B.1*	HSI-MRL	5B	272.33-278.33		
	*Qhsi.hzau-5B.2*	HSI-TRL				
*QHST11*	*Qhsi.hzau-6B.2*	HSI-MRL	6B	231.06-244.73		
	*Qsl.hzau-6B.1*	SL-CK				
*QHST12*	*Qsl.hzau-6B.2*	SL-CK	6B	475.88-485.14		
	*Qhsi.hzau-6B.5*	HSI-MRL				
*QHST13*	*Qhmrl.hzau-7 A*	MRL-HS	7 A	626.631–632.63	*QHttmd.ksu-7 A*	[48]
	*Qhtrl.hzau-7 A*	TRL-HS				
*QHST14*	*Qhsi.hzau-7 A.2*	HSI-MRL	7 A	683.01-689.02	*QSpn.agt-SG.7 A.2*	[20]
	*Qhsi.hzau-7 A.3*	HSI-TRL				
*QHST15*	*Qhmrl.hzau-7D*	MRL-HS	7D	19.58–25.59	*QSLHR.nri-7D*	[36]
	*Qhtrl.hzau-7D*	TRL-HS			*S7D_18808932* (TGW)	[47]
*QRL1*	*Qmrl.hzau-1D*	MRL-CK	1D	485.67-491.67		
	*Qtrl.hzau-1D*	TRL-CK				
*QRL2*	*Qmrl.hzau-7 A*	MRL-CK	7 A	65.55–71.56	*QTwt.agt-L2G.7 A*	[42]
	*Qtrl.hzau-7 A*	TRL-CK				

^a^ QTL-clusters are divided according to the physical distance of stable QTLs

^b^ TKW-DHS, thousands kernel weight under drought and heat stress; GNP-HS, grain number per panicle under heat stress; DM, days to maturity; NDVI, normalized difference vegetation index; MT, membrane thermostability

### Co-localization of QTL and heat shock proteins / heat shock transcription factors

In the 15 QTL-clusters associated with HST, heat shock proteins (HSPs) and heat shock transcription factors (Hsfs) were examined as promising candidates for heat tolerance. Based on the positions of HSPs and Hsfs identified in previous studies [49, 50], a total of 21 HSPs and 1 Hsf were located in 11 HST QTL-clusters (Table 4). Among these, *QHST15* contained the highest number of HSPs. Notably, both *TaHSP100.72* and *TaHSP100.73* were found to be in close proximity to the SNP (SNP-60,804,223) at a distance of 0.23 Mb and 0.33 Mb, respectively. *TaHSP40.121* was located 1.6Kb away from SNP-34,047,520 on Chr4B, which was associated with TRL-HS. *TaHSP100.29* and *TaHSP100.30* were found near SNP-32,700,513 at 84.71 Mb on Chr4B. Additionally, *TaHSP60.35* was located downstream of a SNP on Chr4B associated with MRL-HS, while *TaHSP40.199*, *TaHSP70.103*, and *TaSHSP.134* were found in the vicinity of SNPs on Chr6B and Chr7A, respectively. *TaHsfC1b-1*, near SNP-20,753,978 at 522.01 Mb on Chr3A, was the only heat shock transcription factor detected in this study.

**Table 4:** HSP/Hsf genes associated with QTL-clusters identified in heat stress

QTL-clusters	Chr ^a^	Closest SNP ^b^	Pos (Mb) ^c^	Hsp / Hsf ID ^d^	Gene ID	Distance ^e^
*QHST1*	1 A	SNP-01843442	371.78	*TaHSP60.2*	*TraesCS1A03G0545800*	-2.918
*QHST3*	1D	SNP-08957001	473.35	*TaHSP40.40*	*TraesCS1D03G0936700*	-2.8085
*QHST4*	2 A	SNP-09335596	62.21	*TaHSP70.18*	*TraesCS2A03G0222600*	2.5477
*QHST5*	3 A	SNP-20,753,978	519.86	*TaHsfC1b-1*	*TraesCS3A03G0706100*	1.2642
				*TaHSP40.72*	*TraesCS3A03G0707800*	2.1489
*QHST6*	4B	SNP-32,700,513	84.71	*TaHSP100.29*	*TraesCS4B03G0187800*	0.3012
		SNP-32,700,513	84.71	*TaHSP100.30*	*TraesCS4B03G0188000*	0.3124
		SNP-32,753,382	94.64	*TaSHSP.77*	*TraesCS4B03G0204300*	-0.4468
		SNP-32,756,656	95.12	*TaHSP100.31*	*TraesCS4B03G0205700*	0.4221
		SNP-32,850,736	108.95	*TaHSP40.119*	*TraesCS4B03G0231200*	1.3808
*QHST8*	4B	SNP-33,877,281	348.52	*TaHSP60.35*	*TraesCS4B03G0460900*	-0.228
*QHST9*	4B	SNP-34,047,520	384.81	*TaHSP40.121*	*TraesCS4B03G0494700*	0.0016
*QHST11*	6B	SNP-48,295,383	234.06	*TaHSP40.199*	*TraesCS6B03G0496200*	-0.8988
		SNP-48,295,675	234.10	*TaHSP40.200*	*TraesCS6B03G0499700*	1.2526
*QHST13*	7 A	SNP-55,779,706	629.63	*TaHSP70.103*	*TraesCS7A03G1046300*	-0.665
*QHST14*	7 A	SNP-56,061,347	686.14	*TaSHSP.134*	*TraesCS7A03G1197100*	0.6537
*QHST15*	7D	SNP-60,804,223	22.58	*TaHSP100.72*	*TraesCS7D03G0097800*	0.2262
				*TaHSP100.73*	*TraesCS7D03G0098200*	0.3266
				*TaHSP100.74*	*TraesCS7D03G0102300*	1.1374
				*TaHSP100.75*	*TraesCS7D03G0102800*	1.2187
				*TaHSP100.76*	*TraesCS7D03G0103100*	1.3498
				*TaHSP40.253*	*TraesCS7D03G0104000*	1.8652
				*TaHSP100.77*	*TraesCS7D03G0104400*	1.8975

^a^ Chr, chromosome

^b^ the nearest SNP to *HSP* or *Hsf*

^c^ the physical position of SNP.

^d^ Hsp / Hsf ID referred to Kumar et al. [49] and Ye et al. [50]

^e^ the distance from SNP to *HSP* or *Hsf*; “+” and “-” sign indicates presence of SNP in upstream and downstream

### GO enrichment and expression profiles analysis

**Fig. 5:**
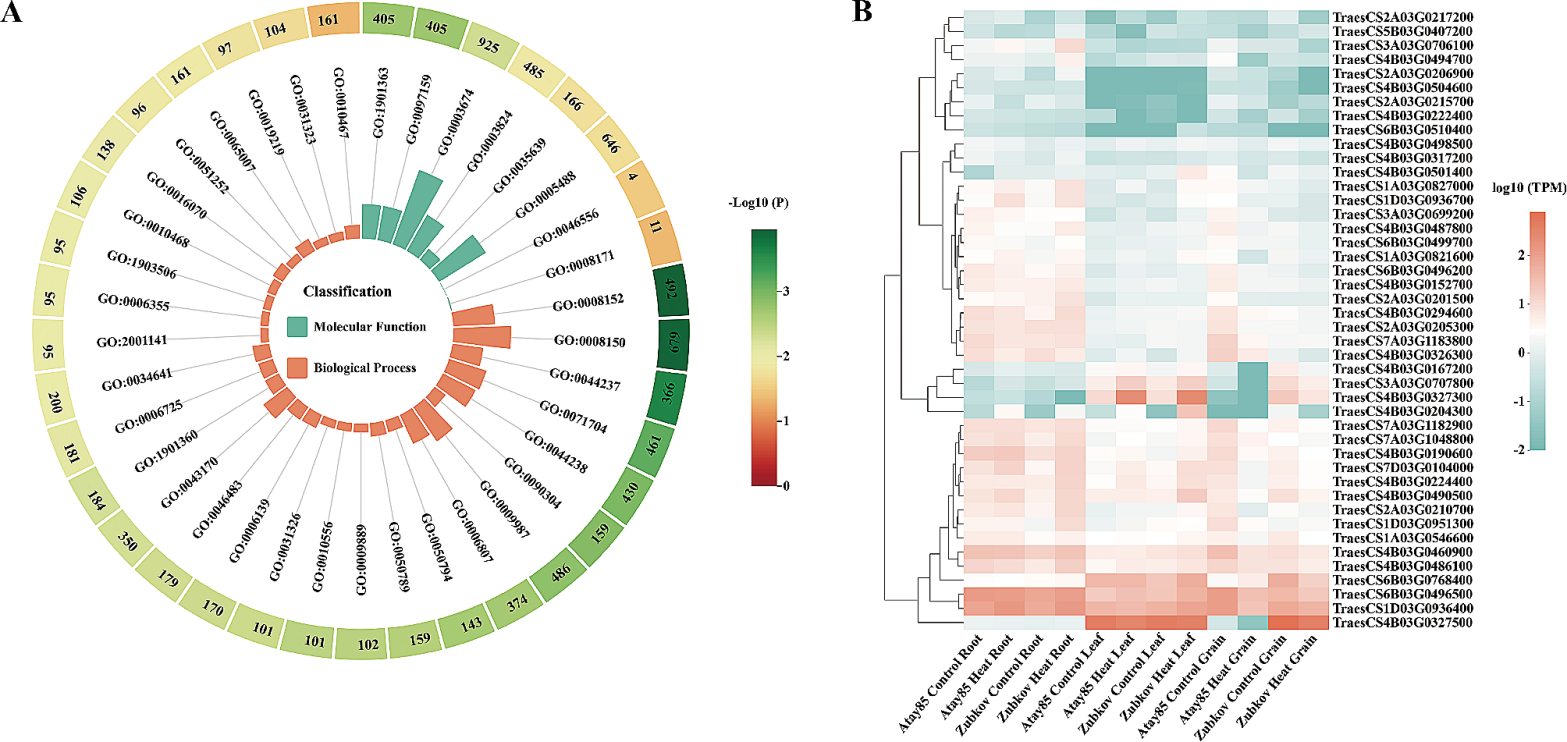
Gene ontology (GO) analysis and expression profiles of the interested genes identified by genome-wide association study (A) GO analysis of 1568 high-confidence genes annotated within the 15 QTL-cluster regions. The first lap indicates number of the genes of each GO term and the significant levels increase from light yellow (the lowest significant level) to dark brown (the highest significant level); the second lap indicates the GO terms and colors denote different classifications. Green and orange mean molecular function and biological process, respectively (B) Heat map diagram shows genes expression patterns of 44 interested genes, including 8 heat shock proteins, 1 heat shock transcription factor and 35 genes associated with abiotic stresses. The genes expression data (accession number: PRJNA358808) were accessed from WheatOmics 1.0 [103]. Expression levels are color-coded green for down-regulated and red for up-regulated. At the right of the diagram the row wise gene ids and at bottom of the tested tissues name are depicted

A total of 1568 high-confidence genes were annotated within the 15 QTL-cluster regions, ranging from 14 genes in *QHST8* to 356 genes in *QHST6* (Table S7). Among the annotated genes, 45.63% (711) were located on chr4B, while only 2.89% (45) were found on chr5B (Fig. S6). Gene ontology (GO) enrichment analysis was performed for these 1568 genes and revealed 37 significant GO terms (Table S8), with 10 terms related to nucleic acid metabolic processes and regulation of transcription, such as nucleic acid metabolic process (GO:0090304), RNA metabolic process (GO:0016070), regulation of RNA metabolic process (GO:0051252), and regulation of RNA metabolic process (GO:0051252), suggesting the impact of heat stress on nucleic acid synthesis and macromolecule metabolism (Fig. 5A).

To narrow down the range of candidate genes associated with HST, homologous genes in *Arabidopsis* and rice were obtained through the BLASTP program. 22 HSPs, 1 Hsf, and 46 genes with annotations related to abiotic stresses were regarded as the promising candidate genes (Table S7). After eliminating genes with little or no expression in all tested tissues, 44 genes were selected for further expression analysis retrieved from public database (http:/202.194.139.32) (Table S9). Most of the 44 genes had a high expression in roots under both normal and heat stress conditions, while the lowest expression levels were observed in leaves (Fig. 5B). In the normal condition, the expression of most genes in the roots (38) and grains (34) of Zubkov, a stress-tolerant wheat variety, were lower than that in Atay-85, a stress-sensitive variety (Fig. 5B; Table S9). Upon increasing temperature, 37 genes were upregulated, and 7 genes were down-regulated in the roots of Zubkov, while 20 genes were upregulated and 24 genes were down regulated in the roots of Atay-85. In the grains, more than 88% of the genes were down-regulated in both Zubkov (39 genes) and Atay-85 (42 genes) after heat stress (Fig. 5B). Although the expression of the majority of the genes in leaves was very low, an increasing trend was observed under heat stress conditions (Fig. 5B). Above all, these genes exhibited different expression patterns in wheat genotypes with different resistance to heat stress. Most of these genes were responsive to high temperature and are worth further analysis.

### Candidate genes for the HST

Among the 44 genes analyzed, 297 SNPs were discovered in 35 genes located within 14 QTL -clusters based on resequencing data (Table S10). These SNPs were distributed as follows: 62.63% in intron and 20.88% in exons (49 synonymous SNPs and 13 missense mutations). The phenotypic differences in heat response between accessions with different alleles at each missense mutation locus were calculated (Table S11; Fig. S7). The results showed that accessions with the AA allele of SNP-32,617,778 in *TraesCS4B03G0152700* exhibited significantly higher heat resistance compared to those with the CC allele (Fig. S7A). Based on the genotype of SNP-32,707,009 in *TraesCS4B03G0190600*, it was shown that AA allele exhibited higher heat resistance (Fig. S7B). For another SNP SNP-32,706,998 in *TraesCS4B03G0190600*, haplotype analysis revealed that the accessions with the reference TT allele fitted a much higher level of heat stress than those with the alternate CC allele (Fig. S7D). Additionally, accessions with the AA allele of SNP-34,070,928 in *TraesCS4B03G0501400* had poor heat resistance, while those with the GG allele showed high heat resistance (Fig. S7C). These results suggested that these three genes are critical genes for HST at the seedling stage in wheat. They are annotated as encoding a WRKY transcription factor (*TraesCS4B03G0152700*; *TaWRKY74-B*), a homeobox leucine zipper protein (*TraesCS4B03G0190600*; *TaHDZ30-4B*), and a non-specific serine/threonine protein kinase (*TraesCS4B03G0501400*; *TaSnRK3.15-B*), respectively, based on previous studies [51–53].

**Fig. 6:**
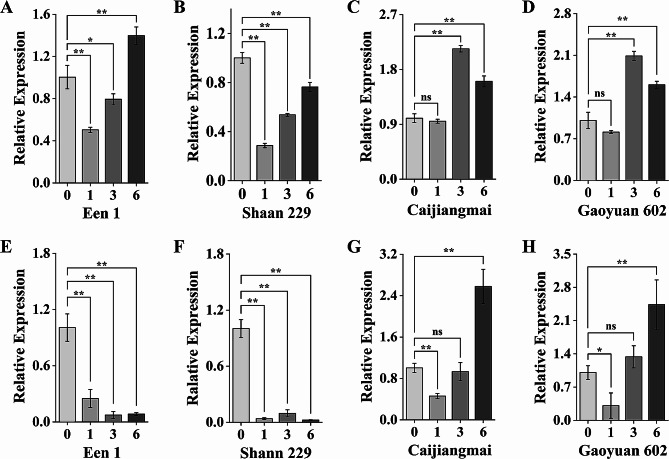
Expression patterns of candidate genes in different seedling tissues under heat condition (A, B, C and D) *TaWRKY74-B* expression profile in Een1, Shaan 229, Caijiangmai and Gaoyuan 602 in roots under heat condition. (E, F, G and H) *TaSnRK3.15-B* expression profile in Een1, Shaan 229, Caijiangmai and Gaoyuan 602 in leaves under heat condition. The quantification of qRT-PCR was calculated using the 2^−ΔΔCt^ method. Bars represent the mean ± SE (standard error) from three replications. The x axis shows 0 h, 1 h, 3 h, and 6 h after transfer the seedling at two-leaf stage from 24℃ to 37℃. * and ** indicate *P* < 0.05 and 0.01, “ns” indicates *P* > 0.05, respectively

To further investigate these critical genes for HST at the seedling stage of wheat, their expression patterns were confirmed in the roots and leaves of the second leaf stage at four-time points (0 h, 1 h, 3 h, and 6 h) under 37℃. This analysis was performed using qRT-PCR in two heat-tolerant varieties (Een 1 and Shaan 229) and two heat-sensitive varieties (Caijiangmai and Gaoyuan602). *TaWRKY74-B* exhibited a similar expression pattern in the roots of the same resistance varieties. In heat-tolerant varieties, *TaWRKY74-B* expression was significantly reduced after one hour 37℃ treatment, followed by a gradually increase in expression (Fig. 6, A-B). In heat-sensitive varieties, no reduced expression was observed after one hour 37℃ treatment. However, the expression level was enhanced after three hours 37℃ treatment, followed by a decrease at six hours 37℃ treatment (Fig. 6, C-D). *TaWRKY74-B* expression was dramatically reduced in leaves of both heat-tolerant and sensitive variety, except for Caijiangmai (Fig. S8, A-D). For *TaSnRK3.15-B*, the expression level in the leaves of heat-tolerant varieties was dramatically down-regulated under heat treatment (Fig. 6, E-F). In contrast, in heat-sensitive varieties, the expression level was slightly decreased and then increased, with the highest values observed at 6 h after the 37℃ treatment (Fig. 6, G-H). The expression level in the leaves seeded to be responsive to temperature and there was no obvious different between two classes of varieties (Fig. S8, E-H). In case of *TaHDZ30-4B*, the expression in the leaves of heat-tolerant varieties was reduced along with the duration of treatment time under high temperature (Fig. S8, I-J). However, it was reversed in one of heat-sensitive varieties Caijiangmai (Fig. S8K), although no expression of *TaHDZ30-4B* was detected in Gaoyuan602 (data not shown). *TaHDZ30-4B* expression in the roots showed no differences between two classes of varieties (Fig. S8, L-N), suggesting that *TaHDZ30-4B* might not be directly relevant to the heat tolerance of wheat.

Taken together, these results indicate that two candidate genes *TaWRKY74-B* and *TaSnRK3.15-B* (Table 5), are likely involved in regulating the physiological and molecular responses of wheat to heat stress.

**Table 5:** Candidate genes of heat tolerance of wheat at seedling stage

QTL-clusters	Name ^a^	Gene ID	Function
*QHST6*	*TaWRKY74-B*	*TraesCS4B03G0152700*	WRKY transcription factor
*QHST9*	*TaSnRK3.15-B*	*TraesCS4B03G0501400*	Non-specific serine/threonine protein kinase

^a^ the nomenclature of *TraesCS4B03G0152700* and *TraesCS4B03G0501400* refers to [51] and [53]

## Discussion

### Phenotypic variation at seedling stage under heat stress

High temperature above 30 °C have a significant impact on the growth and development of wheat [54]. The increasing occurrence of extreme high temperature events poses a major challenge for improving heat resistance in wheat.

While extensive research has been conducted on the effects of heat stress during the adult stages of wheat, such as anthesis, grain filling, and maturation [55–61], relatively few studies have focused on the impact of heat stress during the seedling stage [1, 36, 37, 62–64].

In this study, a diversity panel consisting of 253 wheat accessions was established to characterize the phenotypic variation at the seedling stage under normal and heat stress conditions. The results showed that there were obvious variations among the accessions in terms of SL, RL, TRL under both temperature treatments. The panel was considered suitable for genetic analysis of seedling heat tolerance. The phenotypic data for all traits decreased under heat stress, consistent with previous studies [36, 37]. The analysis also revealed that heat stress had a greater effect on RL compared to SL. Additionally, a significant positive correlation was found between SL under normal conditions and RL under normal conditions, as well as between SL under heat stress and RL under heat stress. These findings suggest that there are common features in the morphological response to heat stress in both shoots and roots, but roots are more susceptible to high temperatures than shoots. These conclusions also support the feasibility of using RL at the seedling stage as an assessment criterion to predict heat tolerance at adult stages.

### Comparisons of the HST QTLs with previously reported

Genetic analysis of heat tolerance in wheat is a hot topic in recent years, and many studies have been performed for heat stress tolerance at flowering and reproductive stages [65–71]. However, there is limited empirical studies focusing on HST at the seedling stage in wheat [1, 36, 72, 73]. In this study, we identified 15 QTL-clusters for HST, which will largely enhance our understanding on heat tolerance and provide valuable genetic information for further QTL fine mapping.

*QHST3* on Chr1D was found to associated with traits such as TRL-HS, MRL-HS, HSI-TRL, and HSI-MRL, which was also identified in previous research (Table 3) [1]. Two QTLs, *QSRHS.nri-2 A* and *QSLHR.nri-7D*, previously found at the seedling stage [36], were also detected in this study shown as *QHST4* and *QHST5* (Table 3). Furthermore, we identified the majority of QTL-clusters (*QHST6*, *QHST7*, *QHST8*, and *QHST9*) on Chr4B, indicating the importance of this chromosome for heat stress tolerance at the seedling stage. Previous studies have reported QTLs related to grain number per spike, days to maturity, normalized difference vegetation index, and membrane thermostability at the adult stage under heat stress in close proximity to the positions of these QTL-clusters (Table 3) [30, 44–47]. In addition, we also observed that many QTLs associated with yield traits under high temperature were located near *QHST1*, *QHST2*, *QHST14*, and *QHST15*, suggesting that some heat stress tolerance mechanisms at the seedling stage may persist throughout the developmental stages of wheat. *QHST8*, *QHST10*, *QHST11*, and *QHST12* were novel for heat stress tolerance as they have never been reported.

Previous reports have indicated that some QTLs can affect a trait under both optimal and stressed conditions [36, 45, 74–76]. Two QTLs on 4B chromosome were shown to regulate shoot length under both heat-stressed and optimal growth condition [36]. In addition, a QTL for thousand kernel weight could be detected on Chr6A under both normal and high temperature stress conditions [74]. In this study, similar findings were observed on chromosomes 1 A, 2 A, and 4B for MRL, TRL, and SL, such as *Qmrl.hzau-1 A* and *Qhmrl.hzau-1 A*, *Qtrl.hzau-4B.1* and *Qhtrl.hzau-4B.1*, *Qsl.hzau-2 A.1* and *Qhsl.hzau-2 A.1*, which were detected under both normal and heat conditions. Therefore, these types of QTLs are crucial for shoot and root growth and could be beneficial in breeding programs aimed at improving crop adaptation to environmental abiotic stresses.

### Candidate genes analysis

Two candidate genes were located on Chr4B. One of these genes, *TaWRKY74-B*, encodes a WRKY transcription factor. WRKY transcription factors play a critical role in plants’ response to abiotic stress [77]. Ectopic expression of another WRKY gene, *TaWRKY75-A* (*TraesCS4A01G193600*), has been shown to improve drought and salt resistances in *Arabidopsis* [49]. Gupta et al. found that many WRKY genes, including *TraesCS1A03G0299200*, *TraesCS2D03G0887300*, and *TraesCS5B03G0589900*, respond to heat stress based on microarray-based analysis [78]. These three genes were confirmed to be heat stress-responsive genes. Another WRKY gene, *TaWRKY33* (*TraesCS6B03G0446800*), localized in the nucleus, greatly increased the survival rate of *TaWRKY33* transgenic Arabidopsis lines after exposure to 45℃ for 5 h [79]. Additionally, it was discovered that *TaWRKY74-4D* (*TraesCS4D03G0129000*) acts as a positive regulator of wheat resistance to *Candidatus* Phytoplasma tritici [80]. Interestingly, the candidate gene *TaWRKY74-B*, was found to be substantially up-regulated under drought conditions in this study [51].

*TaSnRK3.15-B*, a member of the sucrose non-fermenting-1-related protein kinase 3 (SnRK) subfamily, encodes a non-specific serine/threonine protein kinase. SnRK genes, including *TaSnRK* genes, play vital roles in wheat growth and stress responses. Studies have shown that the expression levels of *TaSnRK* genes are significantly regulated under multiple abiotic stresses, including PEG 6000 stress, cold stress, drought stress, heat stress, and phosphorous starvation [53, 81]. This suggests that *TaSnRK* genes are involved in the plant’s response to these stresses. In *Arabidopsis*, overexpression of *TaSnRK2.3* has been found to significantly increase root length and the number of lateral roots, improving stress tolerance under drought, saline, and cold conditions [82]. This indicates that *TaSnRK* genes have the potential to enhance stress tolerance in plants. In addition, Kumar et al. proposed that *SnRK* genes, along with other genes, induce various physiological changes in cells, triggering a complex series of metabolic responses to adapt to hot environments in plants [83]. This highlights the importance of SnRK genes, including *TaSnRK3.15-B*, in plant adaptation to heat stress.

These findings together provide evidence for the involvement of *TaWRKY74-B* and *TaSnRK3.15-B* in the response to high temperature stress and suggest that their expression patterns may contribute to the variation in heat tolerance among wheat varieties. The identification of these candidate genes provides valuable insights into the potential molecular mechanisms underlying heat stress tolerance in wheat and highlights their potential for future breeding efforts to improve heat stress tolerance in crops.

## Conclusions

In this study, 15 QTL-clusters were identified to be associated with heat stress tolerance in wheat at the seedling stage. Based on gene expression, haplotype analysis and gene annotation information within the physical intervals of the 15 QTL-clusters, two candidate genes, *TaWRKY74-B* and *TaSnRK3.15-B*, were successfully predicted as potential regulators of heat stress tolerance. These findings together serve as a fundamental basis for further investigation into the regulatory mechanism underlying high temperature tolerance in wheat.

## Materials and methods

### Plant materials

In this study, a subset of 253 wheat accessions, including 61 landraces and 192 accessions, from various regions around the world were selected as the experimental materials. Among them, 163 accessions were from major agro-ecological zones in China, and 90 accessions were from other countries. Detailed information about each line can be found in Table S1.

### Phenotyping at the seedling stage

A hydroponic growth system was developed using a plastic stent with specific dimensions (50.00 cm × 12.50 cm × 13.00 cm) and 13 grooves (10 cm deep, 45° bank angle). This system allowed for efficient scoring of a large number of varieties. During the experiments, the plastic stent was placed in a box filled with water, and cardboard with wheat seeds was inserted into the grooves of the plastic stent (Fig. S1).

Twelve seeds from each accession were selected and soaked in clean water for 16 h. Six germinating seeds were then chosen and placed in the hydroponic growth system. The experiments were conducted in two growth environments: one mimicking heat stress at 37 ± 1℃ (high temperature) and the other serving as a CK group at 24 ± 1℃ (normal temperature). The other conditions in both growth environments, including illumination intensity (14-hour photoperiod, 200 µmol m^− 2^ s^− 1^) and air relative humidity (70.0 − 75.0%), were consistent in an artificial illumination incubator. Distilled water at the corresponding temperature (37℃ or 24℃) was added to each box to provide water for seedling growth [37]. After 7 days of treatment, the phenotyping images of four seedlings from each of the 253 accessions were taken. The manual process of the EZ-Root-VIS system was used to analyze the phenotyping images and output the main root length (MRL), total root length (TRL), and shoot length (SL) [84]. To detect the roots, (i) the roots less than 30 pixels were rejected, (ii) the roots closer than 3 pixels were merged, and (iii) the terminal roots less than 10 pixels were pruned. The entire experiment was repeated three times, and the average phenotypic data from the three replicates were used for subsequent analysis.

The heat susceptible index (HSI) for different traits was calculated using the formula developed by Fisher and Maurer [85]:

HSI=[1 − Yh/Yp]/H

in which, *Yh* was trait value under heat stress, *Yp* is the trait value under normal condition, *H* = 1 − (mean value of all genotypes under heat stress/mean value of all genotypes under normal condition).

### Statistical analysis

Correlation coefficients (*r*) among all traits were computed using the R software [86]. The package ggplot2 [87] was utilized to generate figures. For basic statistical analysis, two-tailed Student’s *t*-tests, the SAS software version 9.4 (SAS Inc., Cary, N.C., USA) was used.

### DNA extraction and genotyping

High-quality genomic DNA was extracted from fresh leaves of the wheat accessions following the method described by Guillemaut and Laurence [88]. Paired-end sequencing libraries were constructed for each accession according to the manufacturer’s instructions (Illumina, San Diego, CA, USA). The sequencing of all accessions was performed using the MGISEQ-2000 platform.

To analyze the sequencing data, the high-quality reads were aligned to the bread wheat reference genome IWGSC_ref 2.1 [89] using the Burrows-Wheeler Aligner software [90]. SNP calling for each accession was carried out using the Sentieon software [91]. To ensure the quality of the SNP data, SNPs with missing data greater than 20% and minor allele frequency lower than 5% were excluded. The remaining SNPs were used for GWAS to identify genetic regions associated with heat stress tolerance. The SNP annotation was performed based on the reference genome IWGSC_ref 2.1 using SnpEff [92].

The genome sequencing data for the 253 wheat accessions have been deposited in the public database of the China National Genebank (https://db.cngb.org/cnsa) under the accession number CNP0004251 [38, 39].

### Population structure and linkage disequilibrium

Population structure analysis was conducted using the ADMIXTURE software [93]. To determine the optimal *K* value with the smallest cross-validation error (*CV*), five-fold cross-validation was performed for *K* values ranging from 1 to 11, based on polymorphic SNPs. SNPs with an *r*^*2*^ value greater than 0.2 with any other SNP within a 200 kb sliding window were pruned [94]. Principal component analysis (PCA) was performed using Plink version 1.9 to further analyze population structure [95]. A phylogenetic tree was constructed using the neighbor-joining method, with the P distance matrix calculated using VCF2Dis (https://github.com/BGI-shenzhen/VCF2Dis). Visualization of the tree was carried out using tvBOT [96]. The kinship was conducted with Plink and KING software (https://www.kingrelatedness.com/index.shtml), respectively.

Genome-wide pairwise linkage disequilibrium (LD) between SNPs was calculated using PopLDdecay v3.41 [97], and the squared allele frequency correlation (*r*^*2*^) was used as a measure of LD [98].

### Genome-wide association study (GWAS)

GWAS analysis was performed using the factored spectrally transformed linear mixed models (FaST-LMM) [99]. A threshold score of 5.5 (-lg (*P*-value)) was determined to declare significant loci, considering the potential risk of type II error and the GWAS results in this study. The genomic interval 3-Mb upstream and downstream of the significant SNPs were examined as potential QTLs, and overlapping QTLs or QTLs close to each other (distance < 2 Mb) were considered as the same QTLs [38–41]. QTLs with spanned region larger than 50 Mb were excluded [38]. The SNP with the minimum *P*-value was identified as the peak SNP of the QTL based on the *P*-value of the associated SNP [100]. QTLs that were repeatedly detected in at least two traits were selected for subsequent analysis. The naming of the QTLs detected in this research followed the QTL nomenclature guidelines [101].

#### Gene ontology analysis and expression profile

For gene ontology (GO) enrichment analysis, the g: Profiler software was used [102]. GO terms with a false discovery rate (FDR) threshold of 0.05 were considered significant.

To identify genes within the QTL intervals, gene annotations from IWGSC Refseq_2.1 were used. The protein sequences of these genes were aligned with *Arabidopsis* and rice using the BLASTP program to obtain homologous genes in these species. Genes involved in abiotic stress tolerance were identified.

For expression profiling at the tissue level under heat stress treatments, gene expression data for different tissues (leaf, root, and grain) of two cultivars (resistant and susceptible) under normal and heat stress conditions were accessed from WheatOmics 1.0 [103]. The data were used to generate a heatmap for candidate genes using ChiPlot (https://www.chiplot.online/).

### RNA extraction and quantitative real-time PCR (qRT-PCR)

To perform qRT-PCR analysis, seedlings grown to the two-leaf stage in 24℃ condition and transferred to a 37℃ condition. Roots and leaves were collected at 0, 1, 3, and 6 h after heat treatment, immediately frozen in liquid nitrogen, and stored at -80℃. Total RNA was extracted using the *Trelifef™* RNAprep Pure Plant Plus Kit (Tsingke, Beijing, China). Reverse transcription was performed using the Goldenstar RT6 cDNA Synthesis Mix, and qRT-PCR was carried out using the ArtiCanCEO SYBR qPCR Mix. The qRT-PCR profile included an initial denaturation at 95℃ for 1 min, followed by 40 cycles of 95℃ for 15 s, 60℃ for 20 s, and 72℃ for 30 s. A final dissociation stage was performed to generate a melting curve for assessing amplification specificity. The quantification of qRT-PCR was calculated using the 2^−ΔΔCt^ method [104], with the glyceraldehyde-3-phosphate dehydrogenase (GAPDH) gene used as the internal control to normalize the expression levels of the genes of interest. The primers used in qRT-PCR were provided in Table S2.

### Electronic supplementary material

Below is the link to the electronic supplementary material.

Supplementary Material 1

Supplementary Material 2

Supplementary Material 3

Supplementary Material 4

Supplementary Material 5

Supplementary Material 6

Supplementary Material 7

Supplementary Material 8

Supplementary Material 9

Supplementary Material 10

## Data Availability

The datasets generated and analyzed during the current study are available in China National Genebank (https://db.cngb.org/cnsa) under the accession number CNP0004251.

## Abbreviations

*CV*: Cross-validation error
FaST-LMM: Factored spectrally transformed linear mixed models
GAPDH: Glyceraldehyde-3-phosphate dehydrogenase
GO: Gene ontology
GWAS: Genome-wide association studies
HDZ: Homeobox leucine zipper protein
Hsf: Heat shock transcription factor
HSI: Heat susceptible index
HSP: Heat shock protein
HST: Heat stress tolerance
LD: Linkage disequilibrium
MRL: Main root length
MTA: Marker-trait association
PCA: Principal component analysis
QTL: Quantitative trait loci
qRT-PCR: Quantitative real-time PCR
QTN: Quantitative trait nucleotides
RL: Root length
RW: Root weight
SL: Shoot length
SnRK: Sucrose non-fermenting-1-related protein kinase
SW: Shoot weight
TGW: Thousand grain weight
TRL: Total root length
