# Supplementary figures and images for: Genome-wide association study for seedling heat tolerance under two temperature conditions in bread wheat (Triticum aestivum L.)
Source: BMC Plant Biol. 2024 May 21;24:430. doi: 10.1186/s12870-024-05116-2 (PMC11107014; doi:10.1186/s12870-024-05116-2)

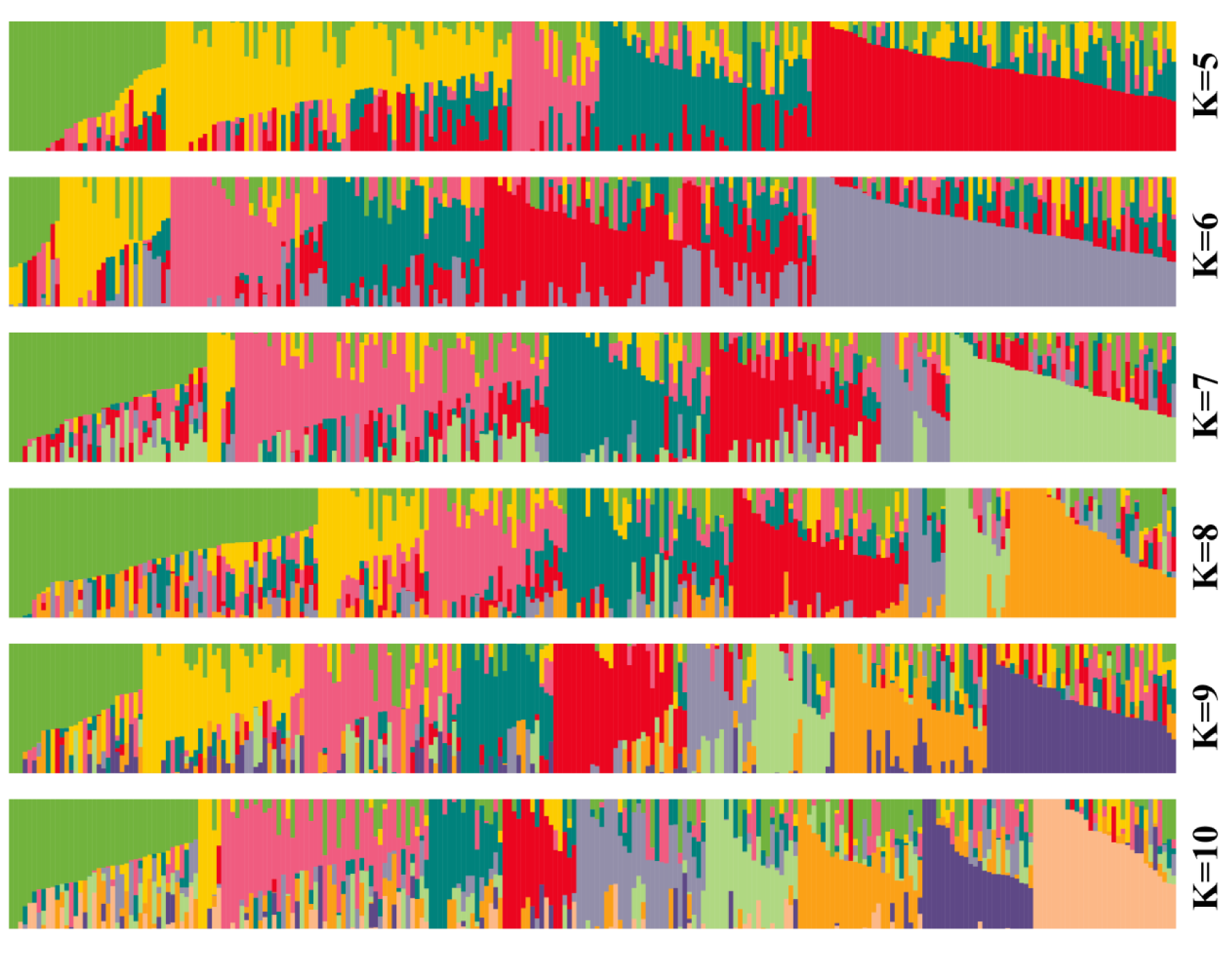

Supplement: Supplementary file 3 — Supplementary Material 3 [file 12870_2024_5116_MOESM3_ESM.png]

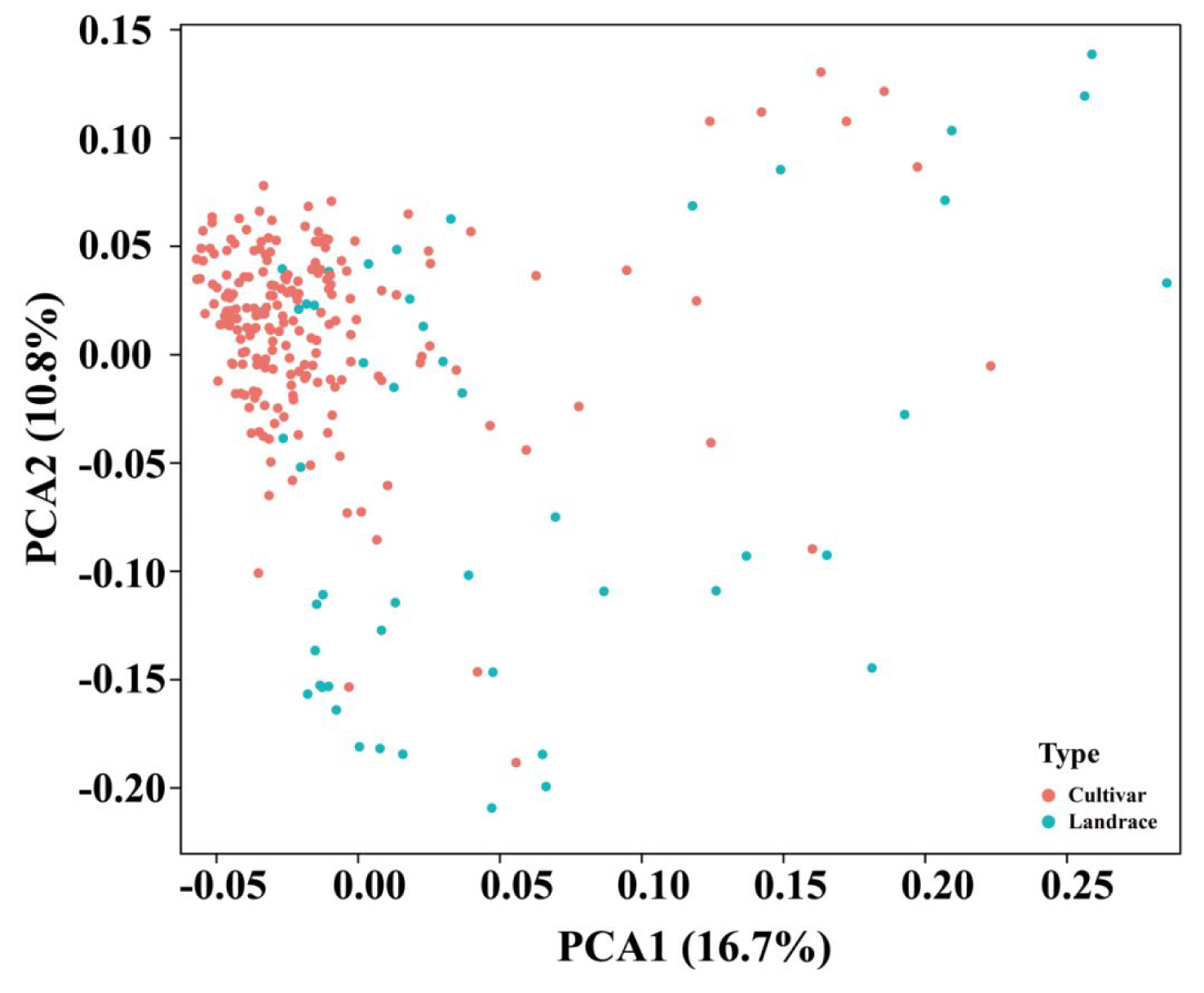

Supplement: Supplementary file 4 — Supplementary Material 4 [file 12870_2024_5116_MOESM4_ESM.png]

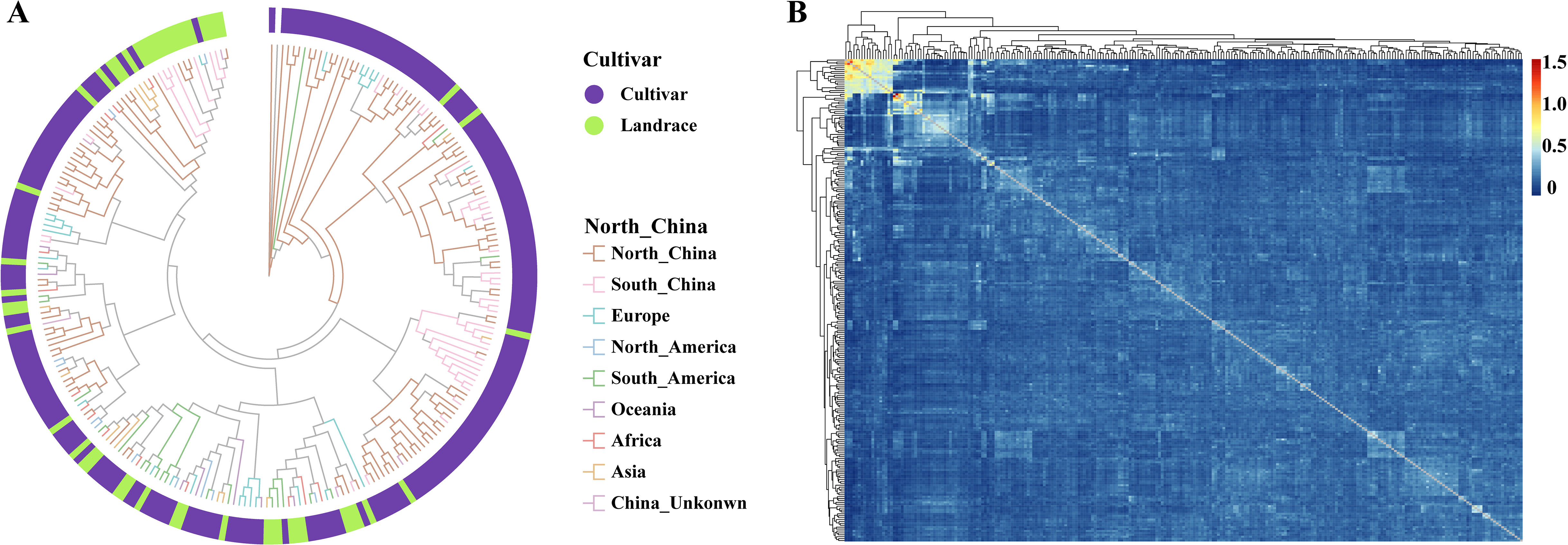

Supplement: Supplementary file 5 — Supplementary Material 5 [file 12870_2024_5116_MOESM5_ESM.png]

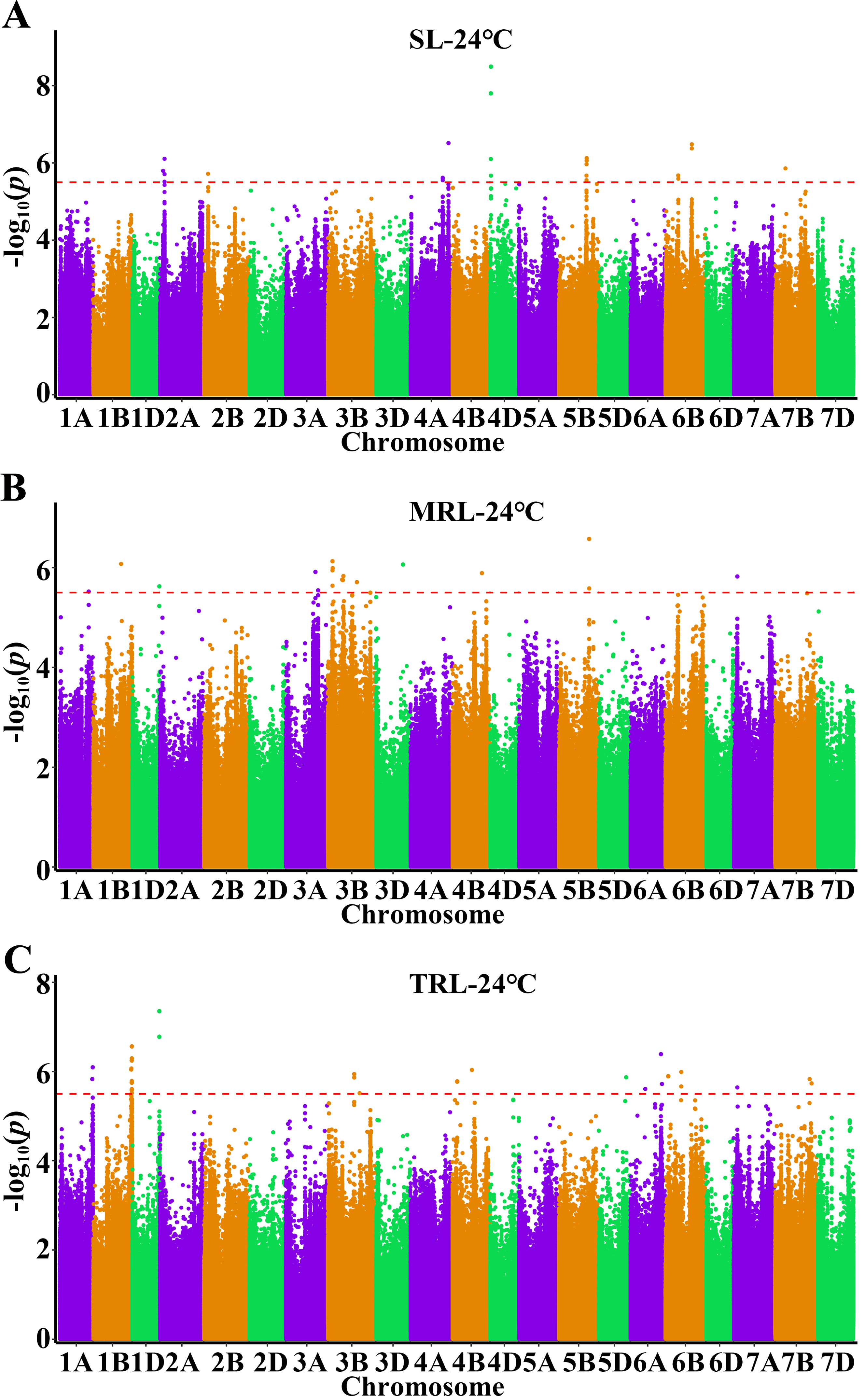

Supplement: Supplementary file 6 — Supplementary Material 6 [file 12870_2024_5116_MOESM6_ESM.png]

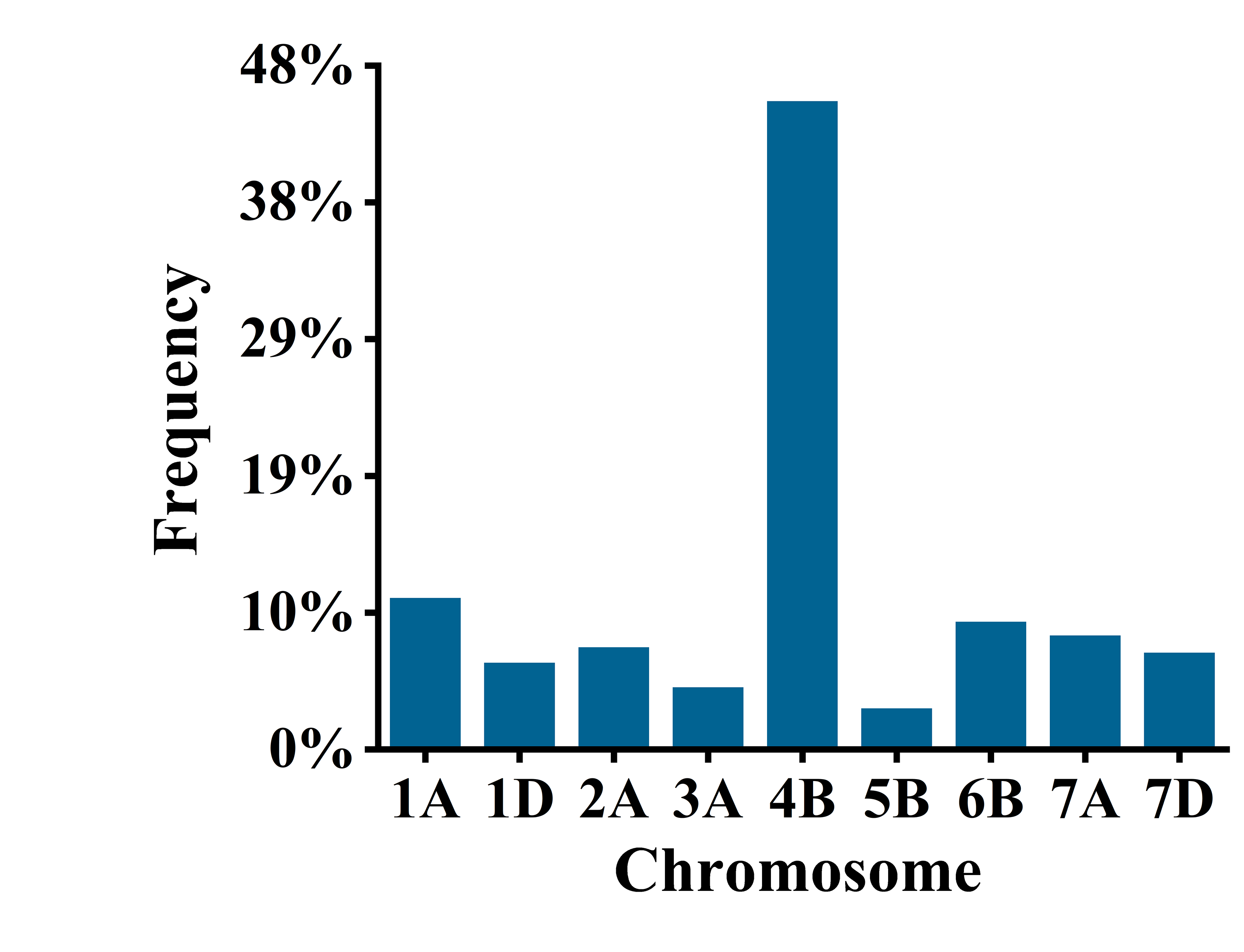

Supplement: Supplementary file 7 — Supplementary Material 7 [file 12870_2024_5116_MOESM7_ESM.png]

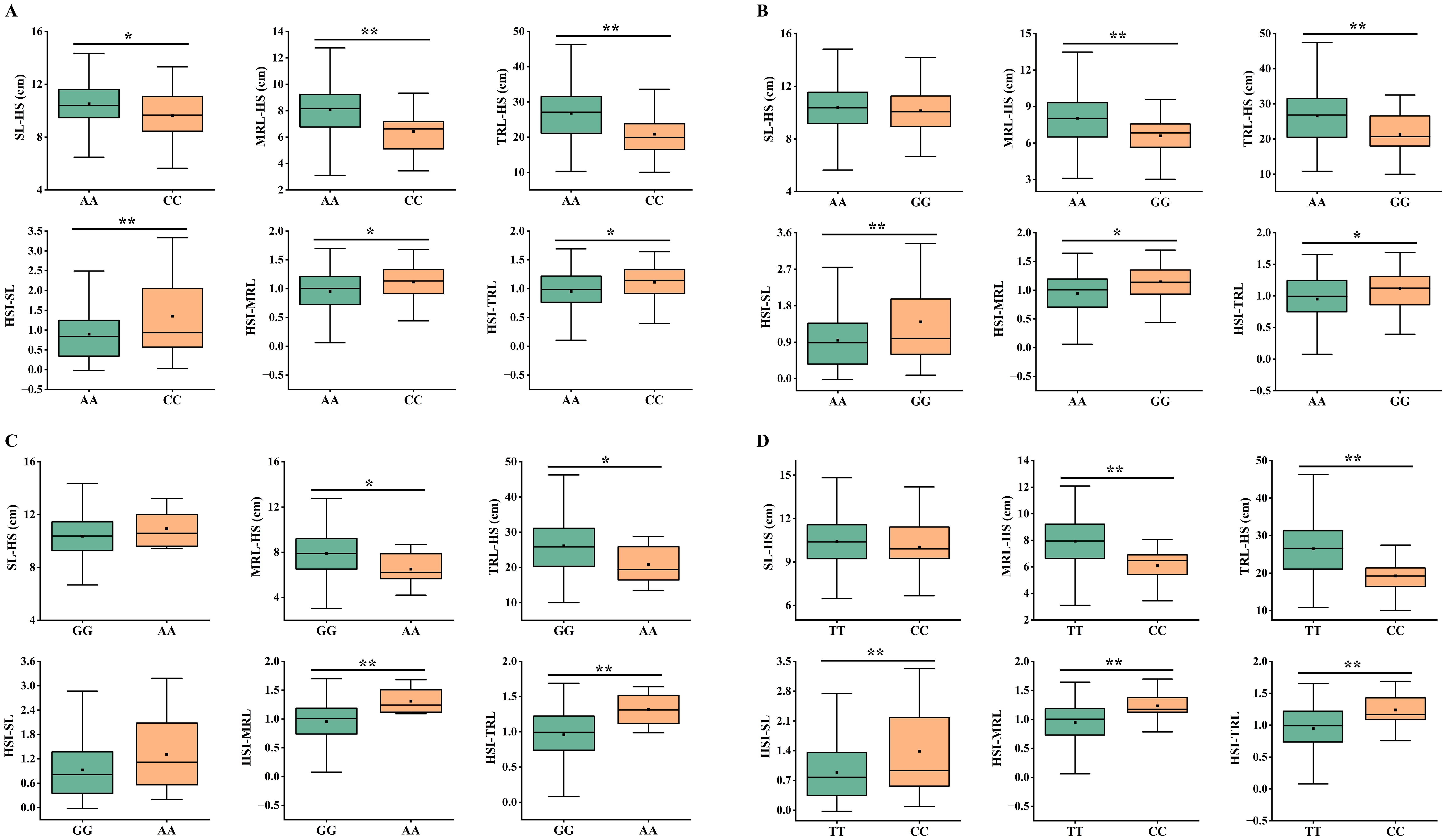

Supplement: Supplementary file 8 — Supplementary Material 8 [file 12870_2024_5116_MOESM8_ESM.png]

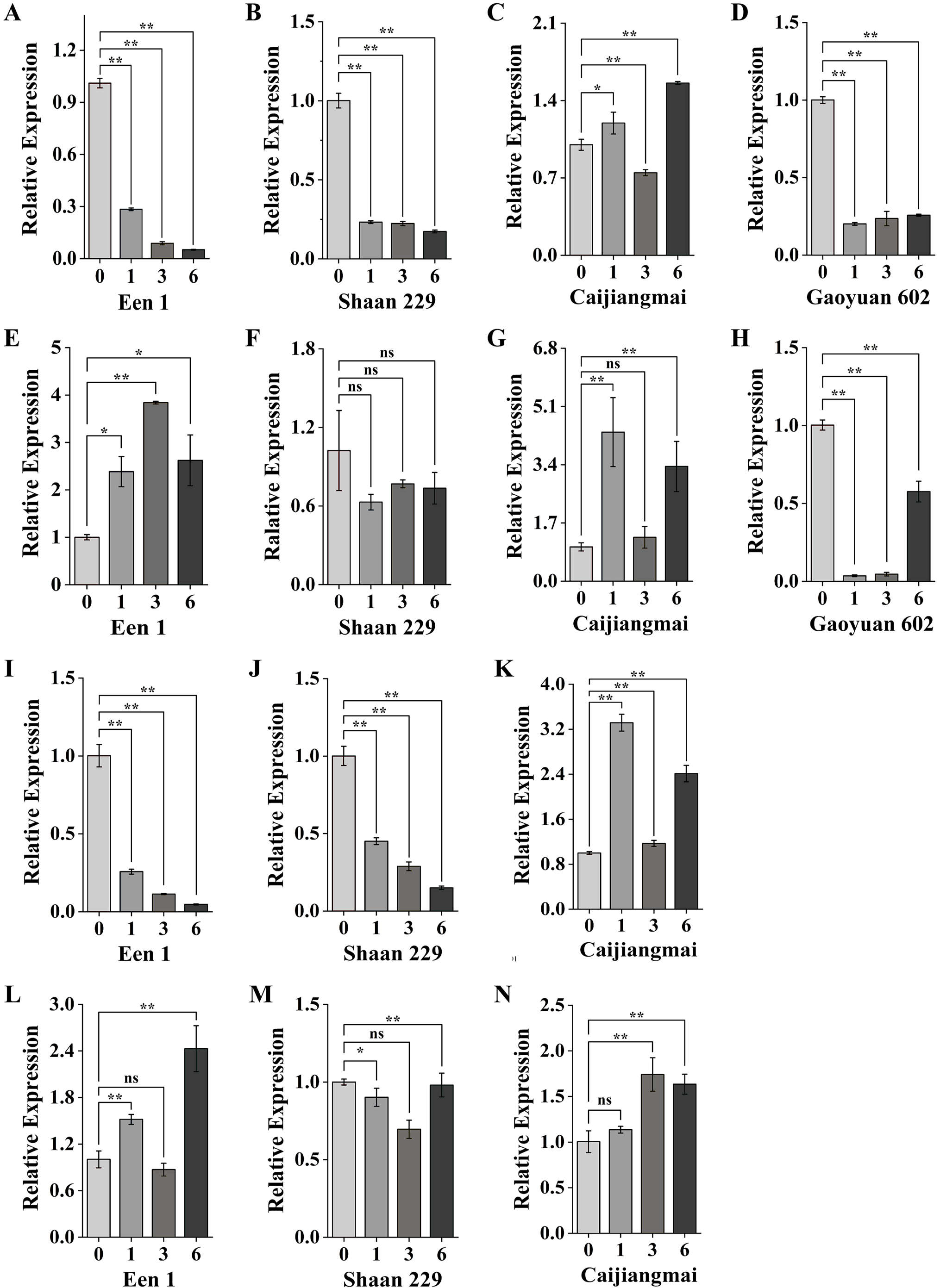

Supplement: Supplementary file 9 — Supplementary Material 9 [file 12870_2024_5116_MOESM9_ESM.png]
