# Supplementary material for: Genome-wide association study for seedling heat tolerance under two temperature conditions in bread wheat (Triticum aestivum L.)
Source: BMC Plant Biol. 2024 May 21;24:430. doi: 10.1186/s12870-024-05116-2 (PMC11107014; doi:10.1186/s12870-024-05116-2)
Supplement: Supplementary file 10 — Supplementary Material 10 [file 12870_2024_5116_MOESM10_ESM.docx]

**Genome-Wide Association Study for seedling heat tolerance under two temperature conditions in bread wheat (*Triticum aestivum* L.)**

**Chao Fu^1^, Ying Zhou^1^, Ankui Liu^1^, Rui Chen^1^, Li Yin^1^, Cong Li^1^, Hailiang Mao^1^***

^1^ National Key Laboratory of Crop Genetic Improvement, Huazhong Agricultural University, Wuhan, 430070, China

*Authors for correspondence: Hailiang Mao: maohailiang@mail.hzau.edu.cn.

**Additional files**

**Additional file 1:**

**Table S1.** Detailed information of 253 lines used in this study.

**Table S2.** Primers used in quantitative real-time PCR.

**Table S3.** High heat tolerance wheat cultivars screened in this study.

**Table S4.** Summary of the distribution of SNPs on 21 chromosomes.

**Table S5.** Summary of significant SNPs associated with all traits in this study.

**Table S6.** All QTLs detected for nine traits in this study.

**Table S7.** All of the high confidence (HC) genes in the QTL-clusters of heat stress.

**Table S8.** The result of GO enrichment in this study.

**Table S9.** Gene expression levels at different treatments and tissues.

**Table S10.** The variants in 35 interested genes.

**Table S11.** Comparison of phenotypes between accessions with favorable and unfavorable alleles at missense mutations loci.

**Figure S1.** Growth system for heat tolerance screening at seedling stage.

This system is space-saving and convenient for carrying out treatment, non-destructive observation and measurement. A plastic stent (50.0 cm × 12.5 cm × 13.0 cm dimension) with 13 grooves (10cm deep, 45° bank angle) was placed in a box containing water, the cardboards with wheat seeds in between were put in grooves of plastic stent.

**Figure S2.** Analysis of the population structure.

Plots of wheat individual ancestry inference for *K* = 5 to 10.

**Figure S3.** Principal component biplot of association population.

Modern cultivars (red dots) and landrace (blue dots) were presented.

**Figure S4.** Neighbour-jointing tree analysis and kinship analyses for the association panel.

(A) Neighbour-jointing tree analysis for the association panel. The first lap indicates the cultivars (purple) and landraces (light green). The colors of branches indicate the geographical origin of wheat lines.

(B) Heatmap of pairwise kinship matrix for evaluating the genetic differences among 253 wheat association. Similarity levels increase from dark blue (the lowest similarity) to dark red (the highest similarity).

**Figure S5.** Manhattan plots of GWAS conducted on three traits of the association mapping panel under 24℃ conditions.

Manhattan plots for shoot length (SL) (A), main root length (MRL) (B) and total root length (TRL) (C) under 24℃ conditions. The x axis represents chromosomes and y axis refers to -log10(*p*) for different traits. Red dash line indicates the threshold of genome-wide significant *P*-value (1×10^−5.5^).

**Figure S6.** Distribution of the high-confidence genes on chromosomes.

**Figure S7.** Box plots of SL, MRL, TRL, and HSI based on the missense SNPs allele.

(A) SNP-32617778 in *TraesCS4B03G0152700*; (B) SNP-32707009 in *TraesCS4B03G0190600*; (C) SNP-34070928 in *TraesCS4B03G0501400*; (D) SNP-32706998 in *TraesCS4B03G0190600*. The significance levels of the differences were analyzed using two-tailed t-test. ** *P* < 0.01 and * *P* < 0.05. SL, shoot length; MRL, main root length; TRL, total root length; HS, heat stress; HSI, heat susceptible index.

**Figure S8.** Expression patterns of interested genes in different seedling tissues under heat condition.

(A, B, C and D) *TaWRKY74-B* expression profile in Een1, Shaan 229, Caijiangmai and Gaoyuan 602 in leaves under heat conditions. (E, F, G and H) *TaSnRK3.15-B* expression in roots of Een1, Shaan 229, Caijiangmai and Gaoyuan 602. (I, J and K) *TaHDZ30-4B* expression in leaves of Een1, Shaan 229, Caijiangmai. (L, M and N) *TaHDZ30-4B* in roots of Een1, Shaan 229, Caijiangmai. The quantification of qRT-PCR was calculated using the 2^-ΔΔCt^ method. Bars represent the mean ± SE (standard error) from three replications. The x axis shows 0h, 1h, 3h, and 6h after the seedling at two-leaf stage in 24℃ conditions transferred to a 37℃ condition. * and ** indicate *P* < 0.05 and 0.01, “ns” indicates *P* ＞ 0.05, respectively.

The expression of *TaHDZ30-4B* was undetectable in Gaoyuan602 and the results were not included here.
